# Next-generation sequencing of the complete mitochondrial genome of the Piao chicken (*Gallus gallus*)

**DOI:** 10.1080/23802359.2020.1791755

**Published:** 2020-07-16

**Authors:** Jingjing Gu, Sheng Li

**Affiliations:** a College of Animal Science and Technology, Hunan Agricultural University, Changsha, China; b Hunan Key Laboratory for Genetic Improvement of Animals, Changsha, China; c Hunan Engineering Research Center of Poultry Production Safety, Changsha, China; d Maxun Biotechnology Institute, Changsha, China

**Keywords:** Piao chicken, mitochondrial genome, next-generation sequencing

## Abstract

In this study, the complete mitochondrial genome sequence of Piao chicken (*Gallus gallus*) was obtained by using the next-generation sequencing method for the first time. The complete mitogenome sequence of Piao chicken contains 2 ribosomal RNAs, 13 protein-coding genes, 22 transfer RNA genes and a D-loop region. This study serves as a valuable genetic resource for mitochondrial studies and can be used in understanding the maternal lineages of native chickens.

Piao chicken is one of the important local chicken breeds in Southwest China. The body shape of Piao chicken looks like a gourd ladle. The chicken has a gentle temperament, strong adaptability to local climate and good meat quality. In recent years, the stocking number of purebreds of Piao chicken is decline rapidly. To investigate the genetic background and improve the breed conservation of Piao chicken, we collected the purebred of this chicken from Pu'er City (22.77 N and 100.97 E), Yunnan province, China. The Piao chicken specimen (Voucher No. P150194) was stored at −80 °C upon collection in the Museum of Hunan provincial key laboratory for genetic improvement of domestic animal, Changsha, China. The extracted total genomic DNA from Piao chicken has been treated as input materials to make sequencing libraries. The sequencing libraries were then sequenced on Illumina Hiseq 2500 sequencer. We obtained 12.45 Gb sequencing reads of Piao chicken in total and deposited those reads in the NCBI Sequence Read Archive (SRA) with accession number SRR4302055. The assembled complete mitochondrial genome of Piao chicken has been submitted to Genbank under accession number MT623706.

The full length of the assembled complete mitochondrial genome of Piao chicken is 16,784 bp. The overall base composition of this mitogenome is 30.2% A, 23.7% T, 32.5% C and 13.5% G, in the order C > A > T > G. The complete mitochondrial genome of Piao chicken has typical circular double strand structure with other vertebrates and contains one control region, 22 transfer RNA genes (tRNAs), 2 ribosomal RNA genes (rRNAs), and 13 protein-coding genes (PCGs). The majority genes (12 PCGs, 2 rRNAs and 14 tRNAs) are encoded on the heavy strand, while others (1PCGs and 8 tRNAs) are encoded on the light strand. All tRNAs are spread among the PCGs and rRNAs. Twelve PCGs initiate with an ATG start codon, except for *COX1* begins with GTG. Four types of stop codons are TAA, TAG, AGG and incomplete codon T– which due to the 5′ terminal of adjacent gene (Anderson et al. 1981). The longest PCG is *ND5*, which is 1818 bp; and the shortest one is the *ATP8*, which is 165 bp. The 12S and 16S ribosomal RNA genes are 977 and 1572 bp in length, respectively. The tRNA genes range from 66 to 76 bp in length.

The phylogenetic position of Piao chicken is inferred by constructed the neighbor-joining (NJ) phylogenetic tree using Mega 7.0 (Kumar et al. 2016) with 1000 bootstrap replicates. The NJ tree (Figure 1) is composed of 38 chicken breeds by using their complete mitochondrial genome sequences. The Piao chicken is closely related with Daweishan Mini, Jianmenguan gray, Hunan indigenous, Nandan and Guangxi Partridge. However, Piao chicken shows the furthest kinship with Wuhua three-yellow. This study serves as a valuable genetic resource for mitochondrial studies and can be used in understanding the maternal lineages of native chickens.

**Figure 1.**
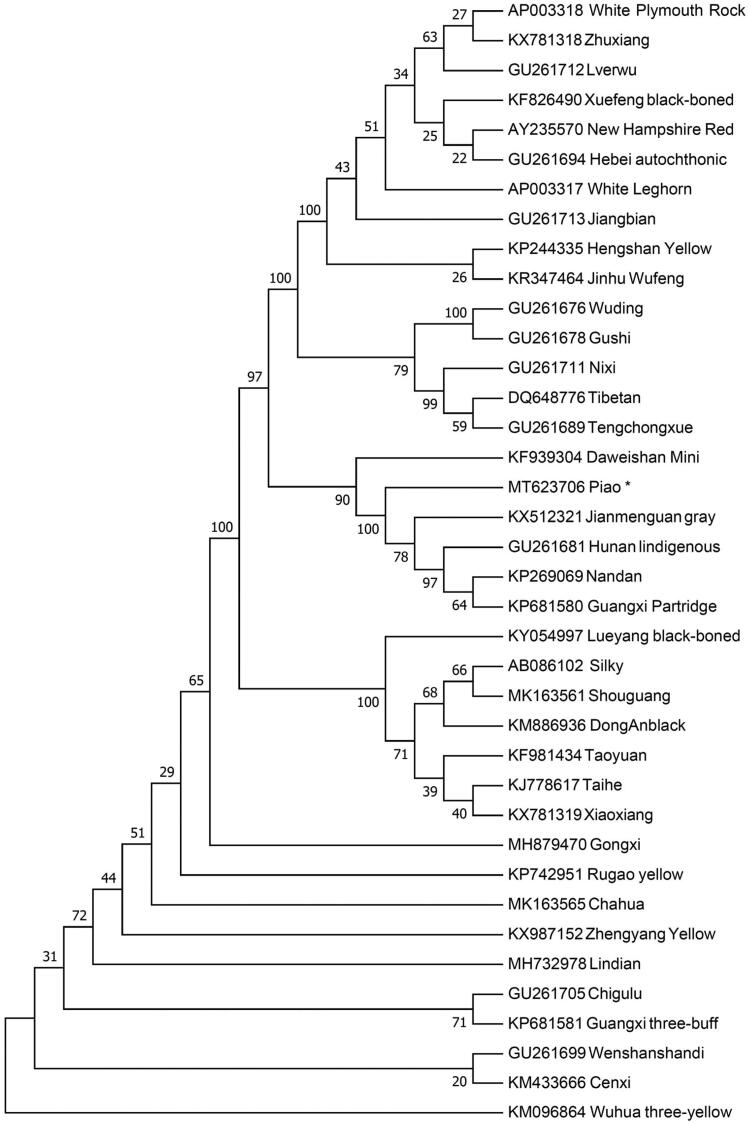
Neighbor-joining tree based on the complete mitochondrial DNA sequence of 38 chicken breeds. GenBank accession numbers are given before the species name.

## Data Availability

The sequence data that support the findings of this study are openly available in the NCBI Sequence Read Archive (SRA) at http://www.ncbi.nlm.nih.gov/sra/ with accession number SRR4302055. The complete mitochondrial genome of Piao chicken (Gallus gallus) is openly available in GenBank at http://www.ncbi.nlm.nih.gov/genbank with accession number MT623706.
